# Does access to clinical study reports from the European Medicines Agency reduce reporting biases? A systematic review and meta-analysis of randomized controlled trials on the effect of erythropoiesis-stimulating agents in cancer patients

**DOI:** 10.1371/journal.pone.0189309

**Published:** 2017-12-11

**Authors:** Eliane Rohner, Michael Grabik, Thomy Tonia, Peter Jüni, Frank Pétavy, Francesco Pignatti, Julia Bohlius

**Affiliations:** 1 Institute of Social and Preventive Medicine, University of Bern, Bern, Switzerland; 2 University of Toronto, Applied Health Research Centre, Li Ka Shing Knowledge Institute, St. Michael's Hospital, Toronto, Ontario, Canada; 3 European Medicines Agency, London, United Kingdom; York University, UNITED KINGDOM

## Abstract

Since 2010, the European Medicines Agency (EMA) has provided access to clinical study reports (CSRs). We requested CSRs for randomized controlled trials (RCTs) of erythropoiesis-stimulating agents (ESAs) in cancer patients from EMA and identified RCT publications with literature searches. We assessed CSR availability and completeness, the impact of unreported and unpublished data obtained from CSRs on the effects of ESAs on quality of life (QoL) of cancer patients, and discrepancies between data reported in the public domain and in CSRs. We used random-effects meta-analyses to evaluate the effect of ESAs on QoL measured with Functional Assessment of Cancer Therapy-Anemia (FACT-An), FACT-Fatigue (FACT-F) and FACT-Anemia Total (FACT-An Total) stratified by data source and the impact of discrepancies on QoL, mortality, adverse events, and clinical effectiveness outcomes. We identified 94 eligible RCTs; CSRs or other study documentation were available for 17 (18%) RCTs at EMA. Median report length was 1,825 pages (range 72–14,569). Of 180 outcomes of interest reported in the EMA documentation, 127 (71%) were publicly available. For 80 of those (63%) we noted discrepancies, but these had little impact on the pooled effect estimates. Of 27 QoL outcomes reported in the CSRs, 17 (63%) were unpublished. Including six unpublished comparisons (pooled mean difference [MD] 0.20; 95% confidence interval [CI] -1.93, 2.33) reduced the pooled effect of ESAs for FACT-An from MD 5.51 (95% CI 4.20, 6.82) in published data to MD 3.21 (95% CI 1.38, 5.03), which is below a clinically important difference (defined as MD ≥4). Effects were similar for FACT-F and FACT-An Total. Access to CSRs from EMA reduced reporting biases for QoL outcomes. However, EMA received documentation for a fraction of all RCTs on effects of ESAs in cancer patients. Additional efforts by other agencies and institutions are needed to make CSRs universally available for all RCTs.

## Introduction

Publication and outcome reporting biases are threats to the validity of evidence synthesis and health care decision-making [1–7]. Relying on published evidence may lead to wrong conclusions about the effects of drug interventions [6,8–12]. Clinical study reports (CSRs) might serve as more complete data sources than publications in medical journals [13–15], and, therefore, assist in the reduction of reporting biases. In general, though, CSRs are not publicly available. However, since 2010, the European Medicines Agency (EMA) has followed a policy on access to documents that affords wider access to documents held by that agency than before [16]. It allows access to CSRs and business-related documents unless there is a need to respect arrangements with regulators outside the European Union (EU) or international organizations, or to protect the privacy and integrity of natural or legal persons.

We requested the EMA retrieve CSRs for studies that evaluated the effect of erythropoiesis-stimulating agents (ESAs) in cancer patients and assessed i) the availability and completeness of CSRs at the EMA, ii) the impact of unreported and unpublished data obtained from EMA documentation on the estimated effects of ESAs on quality of life (QoL), and iii) discrepancies between data for QoL, mortality, adverse events, and clinical effectiveness outcomes reported in the public domain and in the CSRs, and reasons for those discrepancies.

## Methods

This project was conducted under a cooperation agreement between the EMA and the Institute of Social and Preventive Medicine (ISPM) at the University of Bern, Switzerland. All information was treated under conditions of strict confidentiality. The necessary standards of personal data and commercial interest protection were applied in accordance with national and EU legislation. All analyses were done according to a predefined protocol (S1 Protocol). Deviations from the protocol are listed in S1 Text. We included randomized controlled trials (RCTs) that compared ESAs versus placebo or standard treatment in cancer patients receiving or not receiving cancer treatment, who were anemic or at risk of developing anemia. Trial data had to be either available in the public domain or reported in study documentations obtained from the EMA.

### Identification of RCTs submitted to the EMA

We provided a list of International Nonproprietary Names (INN) of products to the EMA. The EMA cross-referenced this list with EMA approved products to identify all the relevant study submissions, both in paper and electronic format. For older submissions, the EMA uses a document-archiving database that is paper based to file the relevant folders. EMA staff used a document-archiving interface to identify clinical modules–the clinical sections of the study submissions–in this database. The relevant sections or tables of contents were then retrieved from the document repository for further evaluation of eligibility at ISPM. Newer submissions were available in electronic format (PDF). Relevant material was transmitted electronically to the review team at ISPM via a secure file-transfer system. At ISPM two reviewers (TT, JB) identified relevant studies from the clinical modules. For eligible studies, the ISPM review team then requested access to all relevant accompanying documents: the CSRs, study protocols including amendments, any appendices, annexes, blank case report forms, and follow-up reports, if available. Two ISPM reviewers (TT, JB) reassessed the eligibility of the identified studies based on the EMA documentation.

### Identification of RCTs in the public domain

We included all RCTs from a previous Cochrane review [17] and updated its literature searches (see S1 Search Strategy for details). For the update, we searched MEDLINE, EMBASE, the Cochrane Central Register of Controlled Trials, conference proceedings (American Society of Clinical Oncology, American Society of Hematology, European Society of Medical Oncology) up to August 2014, and reference lists of relevant systematic reviews [17–20]. Two reviewers (TT, JB) assessed the eligibility of the identified studies. We also searched trial registries, i.e. clinicaltrials.gov and the European Clinical Trials Database (EudraCT), for eligible RCTs.

### Data extraction

Two reviewers (TT, JB) compared documents accessed through the EMA and literature searches to identify documents belonging to the same RCT. One reviewer (ER) extracted data from the EMA documentations using a standardized data extraction form, and a second reviewer (TT) checked the data extractions for accuracy. Similarly, for data in the public domain two reviewers (JB, TT) extracted data from newly identified studies, and we also used the completed data extractions from previous Cochrane Reviews [17–20]. For these Cochrane Reviews, data had been retrieved from publications, personal communication with study authors [18], and reports from the United States Food and Drug Administration (FDA) Oncologic Drugs Advisory Committee hearings. Additionally, individual patient data for mortality and overall survival had been obtained from drug manufacturers and clinical trial study groups [20]. A third reviewer (MG) independently reassessed all data extraction from EMA documentation and a corresponding public domain source.

The data extraction forms included the following items: general study information (title, authors or CSR numbers, trial setting, recruitment dates, funding), trial characteristics (inclusion and exclusion criteria, sample size, baseline characteristics), measures of risk of bias (method of randomization, concealment of allocation, placebo control, adherence to intention-to-treat [ITT] principle), interventions (placebo use, dose, dosing regimen, duration, route of administration, red blood cell [RBC] transfusion trigger, comedications with dose, administration route, and timing), and outcome data. In line with previous Cochrane Reviews [17–20], we assessed the following outcomes of interest: mean difference (MD) in health-related QoL measured with Functional Assessment of Cancer Therapy Fatigue (FACT-F, 13 items) or Functional Assessment of Cancer Therapy Anemia (either using the 20-item scale [FACT-An], or including the general scale of 27 additional items [FACT-An Total]); adverse events, i.e. risk ratios (RRs) for thrombovascular events (TVEs) and hypertension; hazard ratio (HR) for on-study mortality typically defined as mortality during the active study period plus 28 days of follow-up; HR for overall survival during longest follow-up available; and measures of clinical effectiveness, i.e. RR for receiving red blood cell (RBC) transfusions, MD in number of RBC units transfused, MD in hemoglobin change from baseline, and the RR for a hematological response defined as an increase in hemoglobin level of 2 g/dL or more from baseline unrelated to RBC transfusions. Adherence to the ITT principle was defined as less than 10% of randomized patients excluded from the analyses. Disagreements arising at any stage were resolved by discussion and consensus. For information on handling of incompletely reported data see S2 Text.

### Statistical analyses

We used descriptive statistics to assess availability and completeness of CSRs and discrepancies between outcome data reported in the public domain and in EMA documentation. To evaluate the impact of unreported and unpublished data on the pooled effect estimates for QoL outcomes, we meta-analyzed MDs stratified by data source (all available published estimates versus estimates available from EMA documentation only) with random-effects models, and fixed-effects models in predefined sensitivity analyses [21]. If data were available in both the public domain and in the EMA documentation, we chose the data in the public domain for meta-analysis. We compared pooled effect estimates of published and unpublished data using homogeneity tests [21]. For QoL, we defined a clinically important difference as an MD of ≥ 3 for FACT-F [22], ≥ 7 for FACT-An Total [22], and ≥ 4 for FACT-An [23]. We identified discrepancies between outcome data available in the EMA documentation and the public domain by comparing the total number of patients included, the number of events reported, MDs, standard errors, effect estimates, and 95% confidence intervals (CIs). For studies with discrepant outcome data, we used random-effects meta-analysis to pool effect estimates separately for data reported in the EMA documentation and in the public domain. In all meta-analyses, studies with more than one experimental arm were considered as separate comparisons for each experimental arm. We also compared the outcome data for on-study mortality and overall survival as reported in EMA documentation with the results from a previously conducted individual patient data (IPD) meta-analysis [17,24] using random-effects meta-analyses. In a post hoc sensitivity analysis, we merged all experimental arms into a single arm for studies with more than one experimental arm. Analyses were performed using Review Manager 5.1 (The Nordic Cochrane Centre, The Cochrane Collaboration, Copenhagen, Denmark) and Stata 14 (Stata Corporation, College Station, Texas, USA).

## Results

### Studies identified, availability and completeness of clinical study reports

**Fig 1:**
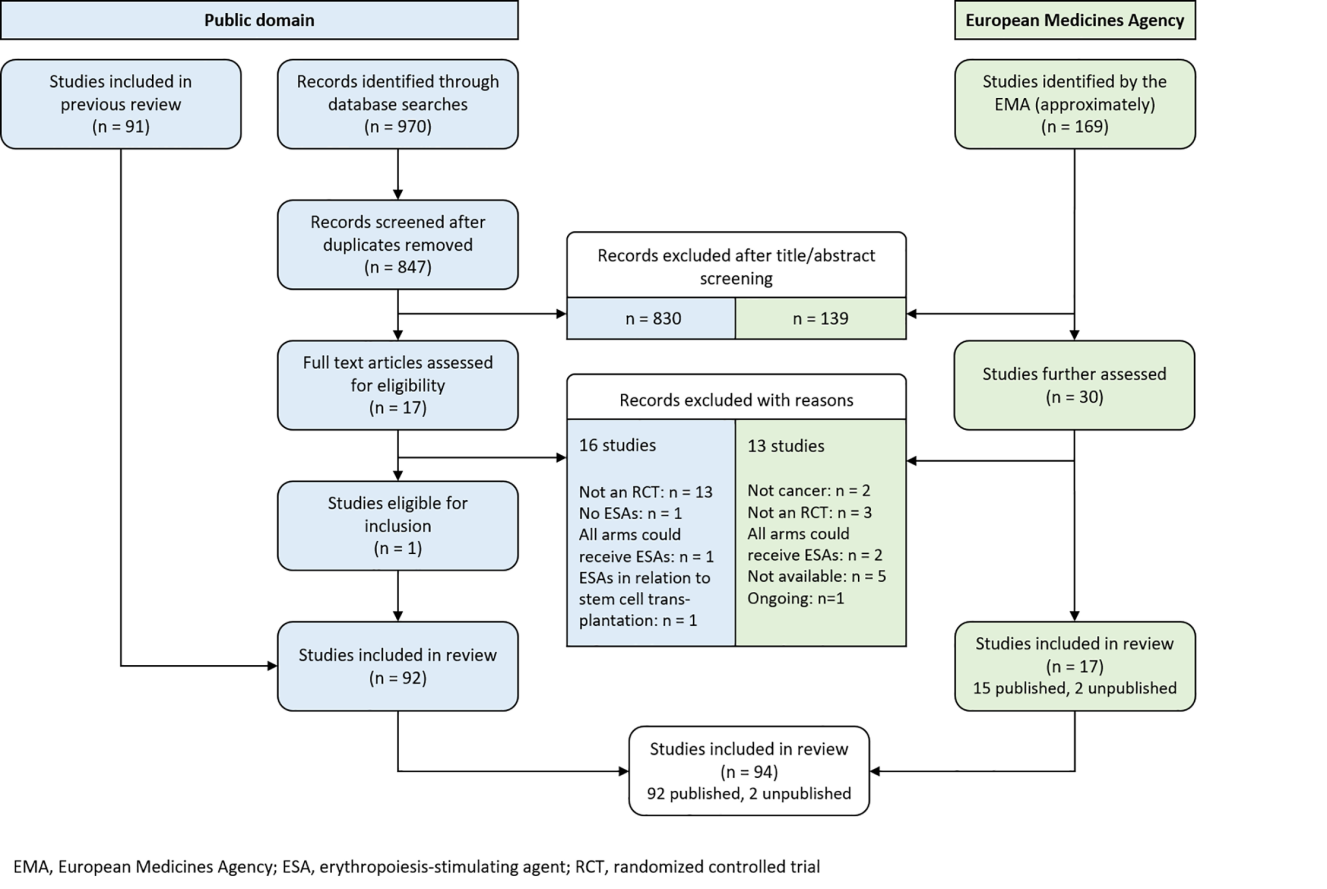
Flow diagram: Identification of studies in the public domain and at the European Medicines Agency.

At the EMA, 169 potentially eligible studies were identified and 30 were assessed in more detail. Seventeen RCTs met our eligibility criteria; 15 of these were published and two were unpublished [25,26]. From literature searches we identified 92 RCTs; no additional RCTs were identified through searches of trial registries. Combining both sources, a total of 94 RCTs were identified and included in the analysis (Fig 1). The EMA provided documentation for 17 RCTs (S1 Table) with a total of 52,287 pages. The median length of the reports was 1,825 pages (range 72–14,569). For 16 RCTs full CSRs were available, for one RCT [27] only a follow-up report was available (CSR number: de-2001-0033).

For most reports, protocols, statistical analyses plans, efficacy and safety evaluations, anonymized individual participant listings for safety and selected efficacy outcomes as well as blank case report forms were available (S2 Table). None of the reports included completed case report forms. Among the 17 RCTs identified at the EMA, six were sponsored by Hoffmann La Roche, four each by Boehringer Mannheim and Amgen, and three by BioGenerix AG.

### Study characteristics

In 66 of the 94 included trials (70%), cancer patients received chemotherapy; study drugs included Darbepoetin (16 trials, 17%) and Epoetin alpha, beta or theta (78 trials, 83%; see Table 1). Half of the studies (47) were placebo controlled. The proportion of RCTs for which random sequence generation was clearly described was similarly low in both the RCTs with and without EMA documentation: 5 of the 17 trials with documentation (29%), and 25 of the 77 without documentation (32%). Allocation was reported to be concealed in 14 RCTs with EMA documentation (82%), and 40 of the RCTs without EMA documentation (52%). In four studies [28–31], either concealment of allocation or sequence generation was judged as adequate based on EMA documentation, and unclear based on the publicly available documents. For one study [32], concealment of allocation was judged as adequate based on the public domain documents, but unclear based on the EMA documentation.

**Table 1:** Characteristics of eligible trials with and without documentation identified at the European Medicines Agency (EMA).

Characteristic		Studies with EMA documentation	Studies without EMA documentation	Total
		N (%)	N (%)	N (%)
**Total**		**17 (100%)**	**77 (100%)**	**94 (100%)**
**Published**	Yes	15 (88%)	77 (100%)	92 (98%)
	No	2 (12%)	0	2 (2%)
**Drug**	Darbepoetin	4 (24%)	12 (16%)	16 (17%)
	Epoetin	13 (76%)	65 (84%)	78 (83%)
**Cancer treatment**	Chemotherapy	14 (82%)	52 (68%)	66 (70%)
	Radiotherapy/Radiochemotherapy	2 (12%)	13 (17%)	15 (16%)
	Unclear/other	0	4 (5%)	4 (4%)
	None	1 (6%)	8 (10%)	9 (10%)
**Design**	1 experimental arm	13 (76%)	70 (91%)	83 (88%)
	≥ 2 experimental arms*	4 (24%)	7 (9%)	11 (12%)
**Sequence generation**	Randomized	5 (29%)	25 (32%)	30 (32%)
	Unclear	12 (71%)	52 (68%)	64 (68%)
**Allocation**	Concealed	14 (82%)	40 (52%)	54 (57%)
	Unclear	3 (18%)	37 (48%)	40 (43%)
**Placebo controlled**	Yes	7 (41%)	40 (52%)	47 (50%)
	No	10 (59%)	37 (48%)	47 (50%)
**Study size**	< 200 participants	7 (41%)	48 (62%)	55 (59%)
	200–400 participants	5 (29%)	24 (31%)	29 (31%)
	> 400 participants	5 (29%)	5 (6%)	10 (11%)

* Studies have one control arm and more than one experimental arm.

### Quality of life outcomes

**Fig 2:**
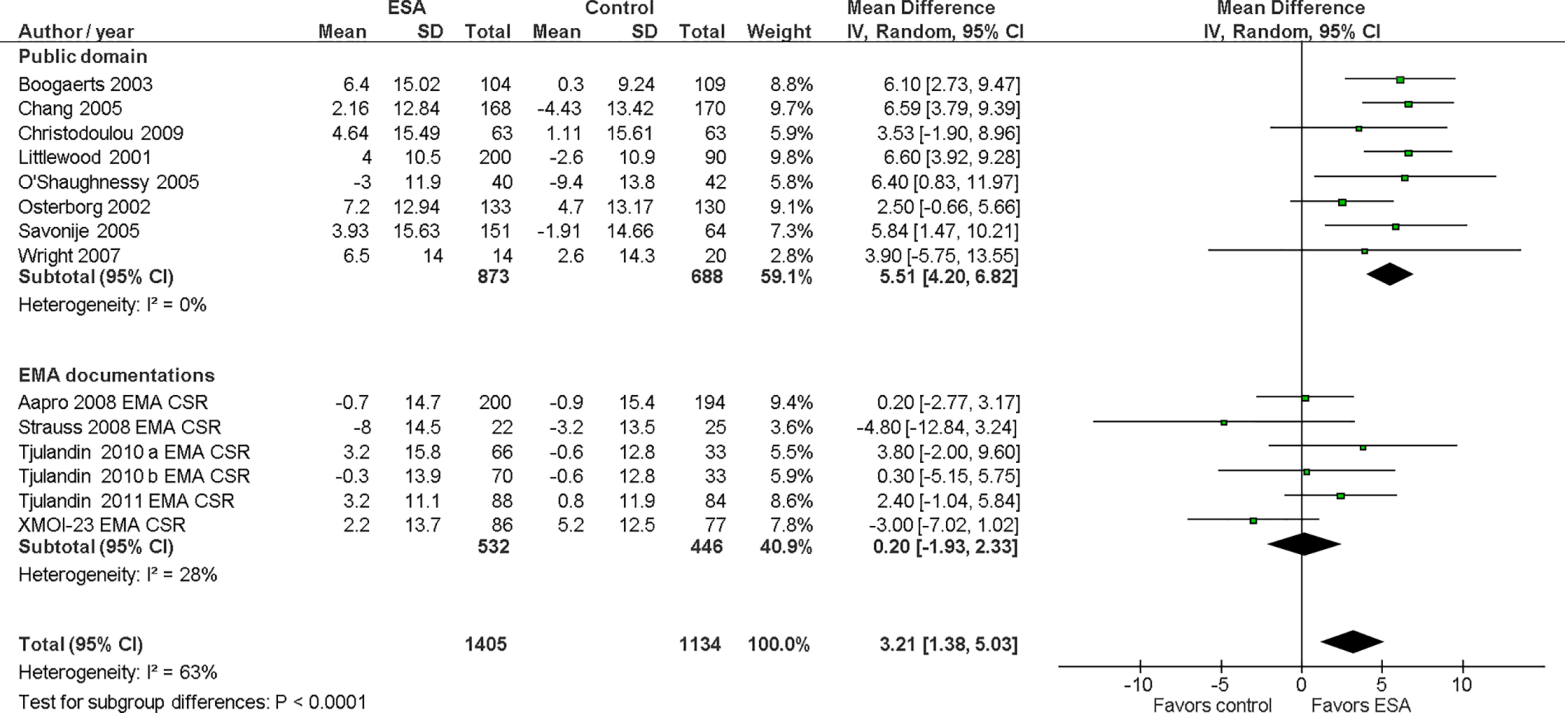
FACT-An stratified by source of data: public domain versus EMA documentation. CI, confidence; EMA, European Medicines Agency; ESA, erythropoiesis-stimulating agents; FACT-An, Functional Assessment of Cancer Therapy-Anemia; IV, inverse variance; SD, standard deviation.

Ten of the 17 RCTs with EMA documentation (59%) and 24 of the 92 published RCTs (26%) reported QoL related outcomes (FACT-F, FACT-An, and FACT-An Total). In total, 27 QoL outcomes were reported in the RCT documentation from the EMA and 37 in the published RCTs. Ten of the 27 QoL outcomes reported in the EMA documentation (37%) were also available in the public domain resulting in 54 QoL outcomes overall. Based on published data, the pooled MD for the effect of ESAs on QoL in cancer patients measured with FACT-An was 5.51 (95% CI 4.20, 6.82; 8 comparisons, n = 1,561). The effect of ESAs on FACT-An for the six unpublished comparisons reported in EMA documentation was MD 0.20 (95% CI -1.93, 2.33). The 95% CIs of the pooled effect estimates from published and unpublished comparisons for FACT-An did not overlap (p-value for interaction <0.001; see Fig 2). Including the unpublished data from the EMA documentation reduced the pooled estimate to MD 3.21 (95% CI 1.38, 5.03; 14 comparisons, n = 2,539), which is below the threshold of a clinically important difference for FACT-An (defined as MD ≥4). For FACT-F, including data from EMA documentation reduced the pooled estimate from MD 2.37 (95% CI 1.40, 3.35; 18 comparisons, n = 4,965) to MD 1.93 (95% CI 1.04; 2.83; 23 comparisons, n = 5,771). The pooled effect of ESAs on FACT-F for the five unpublished comparisons alone was MD -0.12 (95% CI -1.63, 1.38). The 95% CIs of the pooled effect estimates from published and unpublished comparisons for FACT-F did not overlap (p-value for interaction = 0.006; see S1 Fig). Effect estimates from both published and unpublished comparisons were below the threshold of a clinically important difference (defined as MD ≥3). For FACT-An Total, 11 published (pooled MD 5.97, 95% CI 0.49, 11.44) and six unpublished comparisons (pooled MD -0.17, 95% CI -4.82, 4.48) were included (p-value for interaction = 0.09; S2 Fig). Effect estimates from both published and unpublished comparisons for FACT-An Total were below the threshold of a clinically important difference (defined as MD ≥7). The pooled effect estimates remained similar when we used fixed-effects models instead of random-effects models (S3 Table), and when we merged experimental arms from multi-arm studies (S4 Table).

### Discrepancies between outcome data

In the EMA documentation available for 17 RCTs, a total of 180 outcomes of interest were reported that encompassed QoL, adverse events, mortality, overall survival, and measures of clinical effectiveness. Among these, 127 (71%) were already available in the public domain. Eighteen of the 53 outcomes that were not available in the public domain belonged to two unpublished RCTs [25,26], while the other 35 outcomes belonged to 10 published studies that did not report these outcomes in primary or secondary sources (14 QoL outcomes, 18 adverse events outcomes, and 3 clinical effectiveness outcomes). For 80 of the 127 outcomes available both in the public domain and in the EMA documentation (63%) we noted discrepancies, but these discrepancies had little impact on the pooled effect estimates (S5 Table). For example, TVEs were generally reported as composite endpoints in the public domain and as components of composite endpoints in the EMA documentation. Therefore, estimates were often not directly comparable. The total numbers of TVEs reported in the public domain and in the EMA documentation differed substantially for some studies [30,31,33–35]. However, the pooled relative effect estimates for studies with discrepant data were similar in both the public domain (RR 1.52, 95% CI 1.09, 2.13) and in the EMA documentation (RR 1.22, 95% CI 0.94, 1.58). Comparing overall survival and mortality from public domain with data from EMA documentation, we noticed some differences in the numbers of patients and deaths included in analyses. These discrepancies were partly due to differences in the length of follow-up for overall survival, or differences in the definition of the end of study for mortality. Comparing overall survival and mortality from IPD analyses with estimates reported in EMA documentation, we noticed some differences in the number of patients and deaths included in analyses, however, pooled estimates were similar (S6 Table). For 65 of the 127 outcomes (51%) results were reported in adherence with the ITT principle in the EMA documentation and in the public domain; for 56 of outcomes (44%) results were reported not in adherence with the ITT principle in EMA documentation and in the public domain. For six outcomes from three studies [31,34,36], ITT data were discrepant with either ITT analyses being reported in the EMA documentation but not in the public domain (4 trials), or vice versa (2 trials). All of these outcomes were continuous, and the discrepancies were explained by using either the last observation measured in the analyses, or the last-observation-carried-forward method.

## Discussion

For only 18% of RCTs included in our study were CSRs or follow-up reports available at the EMA. Although the number of trials with RCT documentation identified at the EMA was small, it included two unpublished studies and several unreported QoL outcomes. Unpublished and unreported QoL outcomes tended to show smaller effects of ESAs than published QoL outcomes. Including these unpublished and unreported data from EMA documentation reduced the pooled estimates for the effect of ESAs on the anemia-related symptoms of cancer patients, measured with FACT-An, from MD 5.51 to MD 3.21, which is below the threshold of a clinically important difference. Discrepancies between outcome data reported in the public domain and in the EMA documentation were common, but they had little impact on the pooled effect estimates.

The various strengths and limitations of our study are as follow. We obtained RCT documentation from the EMA and evaluated a set of predefined outcomes. We based our study on a long-standing review activity and had previously retrieved unpublished data for clinical effectiveness outcomes [18], adverse events, mortality, and survival [20,24]. Available now in secondary sources [18,20,24],these data allowed us to assess discrepancies for a large number of outcome data. However, these data may not be representative for outcome data that are typically available in the public domain. Thus, we used QoL outcomes, for which no additional unpublished and unreported data were available, to assess the impact of access to EMA documentation on reporting biases. But the different QoL scales we assessed (FACT-An, FACT-F, and FACT-An Total) are interrelated and may therefore not represent independent evidence for reporting biases. As reporting biases was the focus of our study, we did not consider risk of bias or other study and patient characteristics in our analyses.

The two unpublished studies and several unreported outcomes we identified in EMA documentation confirm previous findings showing that unpublished and unreported data can be obtained from CSRs [10–14,23]. Our inclusion of unpublished comparisons and the reduction of the pooled estimates for the effect of ESAs on QoL outcomes further confirm previous studies that also have shown that unpublished and unreported data tend to show less favorable effect estimates than published data [12–14,23], and that the inclusion of unpublished and unreported data may change the pooled effect estimates for a given outcome [12–14,23]. The QoL estimates in our study were more conservative compared to previous meta-analyses [17,23,37–39]. However, our QoL data are still incomplete. For the analysis of FACT-An we included 14 comparisons from 13 RCTs. A previous meta-analysis [23] identified another 18 RCTs measuring anemia-related symptoms using FACT-An, but these data are neither available in the public domain nor at the EMA. With a large proportion of RCT data still missing, the true effect of ESAs on QoL remains uncertain. We identified discrepancies between RCT documentation obtained from the EMA and publicly available outcome data, but these discrepancies had no or little impact on the pooled effect estimates. This finding is in contrast with previous studies that found relevant discrepancies between CSRs and the public domain and highlighted the need to scrutinize the data carefully [40].

The EMA has granted access to CSRs of drugs since 2010 [16]. However, not all drug trial results within Europe are required to be sent to the EMA and for only a fraction of the included RCTs were CSRs and follow-up reports available at the EMA. In addition, not all drugs are licensed in Europe, and the EMA has no authority outside of Europe. To overcome these limitations, efforts of the FDA [41] and other agencies and institutions are needed to make CSRs universally available for all trials.

## Conclusions

Access to CSRs requested from the EMA can reduce reporting biases. However, the EMA receives CSRs only for RCTs that are part of EU regulatory submissions, and those received are only a fraction of all RCTs on the effect of ESAs in cancer patients. Additional efforts by other agencies and institutions are needed to make CSRs universally available for all RCTs.

## Supporting information

S1 Checklist PRISMA checklist. (DOC)

S1 Dataset Study characteristics of included randomized controlled trials. (XLS)

S2 Dataset Outcome data stratified by source of data and by discrepancies. (XLS)

S1 Fig FACT-F stratified by source of data: Public domain versus EMA documentation only. CI, confidence interval; EMA, European Medicines Agency; ESA, erythropoiesis-stimulating agent; FACT-F, Functional Assessment of Cancer Therapy-Fatigue; IV, inverse variance; SD, standard deviation.
(TIF)

S2 Fig FACT-An Total stratified by source of data: Public domain versus EMA documentation only. CI, confidence interval; EMA, European Medicines Agency; ESA, erythropoiesis-stimulating agent; FACT-F, Functional Assessment of Cancer Therapy-Fatigue; IV, inverse variance; SD, standard deviation.
(TIF)

S1 Search strategy Literature search in MEDLINE. (DOCX)

S1 Study protocol Does access to clinical study reports from the European Medicines Agency reduce reporting bias? (DOCX)

S1 Text Protocol changes. (DOCX)

S2 Text Handling of incompletely reported outcome data. (DOCX)

S1 Table Characteristics of eligible trials identified at the European Medicines Agency (EMA). CLL, chronic lymphocytic leukemia; MM, multiple myeloma; NHL, non-Hodgkin lymphoma; ODAC, Oncologic Drugs Advisory Committee; pc, personal communication; Q2W, every second week; Q3W, every third week; Q4W, every fourth week; sc, subcutaneous; TIW, three times per week.
(DOCX)

S2 Table Completeness of RCT documentation provided by the European Medicines Agency. (DOCX)

S3 Table Meta-analyses for quality of life stratified by source of data. CI, confidence interval; FACT-An, Functional Assessment of Cancer Therapy-Anemia; FACT-F, Functional Assessment of Cancer Therapy-Fatigue; MD, mean difference.
(DOCX)

S4 Table Meta-analyses for quality of life stratified by source of data: Sensitivity analyses with merged experimental arms for multi-arm studies. CI, confidence interval; FACT-An, Functional Assessment of Cancer Therapy-Anemia; FACT-F, Functional Assessment of Cancer Therapy-Fatigue; MD, mean difference.
(DOCX)

S5 Table Direct comparison of pooled effect estimates from studies that reported discrepant data in the public domain and in EMA documentation. CI, confidence interval; EMA, European Medicines Agency; FACT-An, Functional Assessment of Cancer Therapy-Anemia; FACT-F Functional Assessment of Cancer Therapy-Fatigue; Hb, hemoglobin; HR, hazard ratio; MD, mean difference; RBC, red blood cell; RR, risk ratio.
(DOCX)

S6 Table Comparison of pooled effect estimates from studies where individual patient data were used for the analysis of on-study mortality and overall survival in previous analyses and estimates reported in EMA documentation. CI, confidence interval; EMA, European Medicines Agency; HR, hazard ratio.
(DOCX)

## Abbreviations

CSR: clinical study report
CI: confidence interval
EMA: European Medicines Agency
ESA: erythropoiesis-stimulating agent
FACT-An: Functional Assessment of Cancer Therapy-Anemia
FACT-An Total: Functional Assessment of Cancer Therapy-Anemia Total
FACT-F: Functional Assessment of Cancer Therapy-Fatigue
MD: mean difference
RCT: randomized controlled trial
QoL: quality of life
