# Supplementary figures and images for: Does access to clinical study reports from the European Medicines Agency reduce reporting biases? A systematic review and meta-analysis of randomized controlled trials on the effect of erythropoiesis-stimulating agents in cancer patients
Source: PLoS One. 2017 Dec 11;12(12):e0189309. doi: 10.1371/journal.pone.0189309 (PMC5724886; doi:10.1371/journal.pone.0189309)

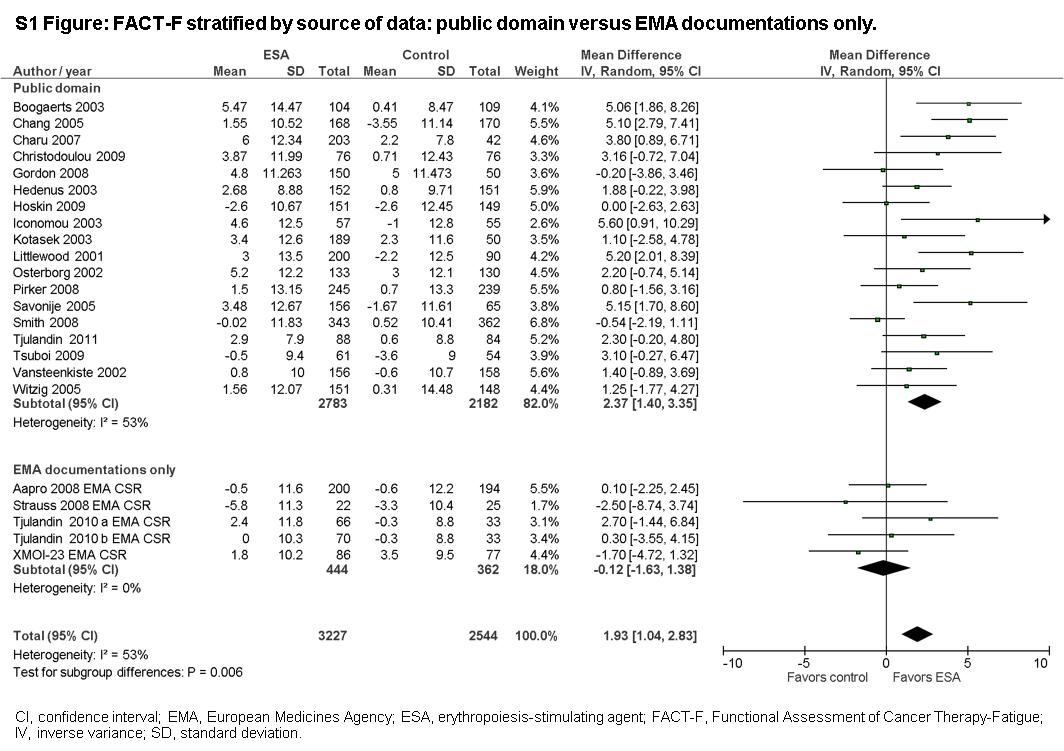

Supplement: S1 Fig — CI, confidence interval; EMA, European Medicines Agency; ESA, erythropoiesis-stimulating agent; FACT-F, Functional Assessment of Cancer Therapy-Fatigue; IV, inverse variance; SD, standard deviation. (TIF) [file pone.0189309.s004.tif]

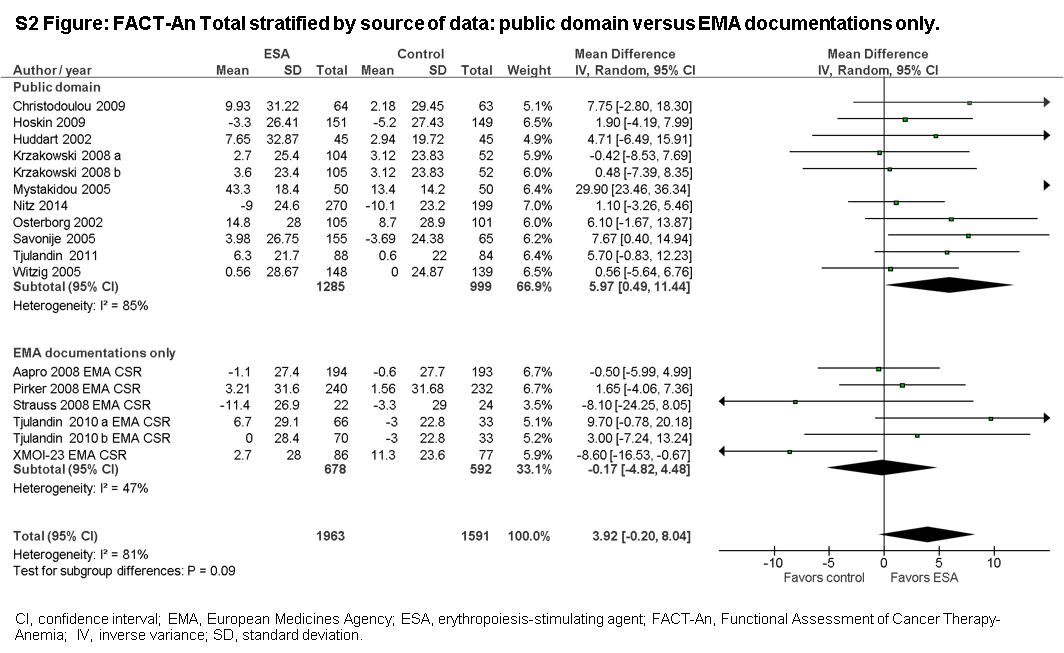

Supplement: S2 Fig — CI, confidence interval; EMA, European Medicines Agency; ESA, erythropoiesis-stimulating agent; FACT-F, Functional Assessment of Cancer Therapy-Fatigue; IV, inverse variance; SD, standard deviation. (TIF) [file pone.0189309.s005.tif]
