# Supplementary material for: Does access to clinical study reports from the European Medicines Agency reduce reporting biases? A systematic review and meta-analysis of randomized controlled trials on the effect of erythropoiesis-stimulating agents in cancer patients
Source: PLoS One. 2017 Dec 11;12(12):e0189309. doi: 10.1371/journal.pone.0189309 (PMC5724886; doi:10.1371/journal.pone.0189309)
Supplement: S1 Search strategy — (DOCX) [file pone.0189309.s006.docx]

**S1 Search strategy**

The literature search was performed without any restrictions concerning publication status or language. For example, the literature search in MEDLINE was as follows:

| **#** | **Searches** |
| --- | --- |
| 1 | exp ERYTHROPOIETIN/ |
| 2 | erythropoietin.mp. |
| 3 | erythropoiesis.mp. |
| 4 | epoetin.mp. |
| 5 | epo.mp. |
| 6 | epoetin alfa.mp. |
| 7 | epoetin beta.mp. |
| 8 | eprex.mp. |
| 9 | neorecormon.mp. |
| 10 | aranesp.mp. |
| 11 | procrit.mp. |
| 12 | recombinant erythropoietin.mp. |
| 13 | darbepoetin alfa.mp. |
| 14 | darbepoetin.mp. |
| 15 | RECEPTORS, ERYTHROPOIETIN/ |
| 16 | CERA.mp. |
| 17 | or/1-16 |
| 18 | exp ANEMIA/dt, th [Drug Therapy, Therapy] |
| 19 | anaemia.mp. |
| 20 | anemia.mp. |
| 21 | (anemi$ adj3 cancer).mp. |
| 22 | (anaemi$ adj3 cancer).mp. |
| 23 | or/18-22 |
| 24 | exp Neoplasms/ |
| 25 | malignan$.mp. |
| 26 | cancer$.mp. |
| 27 | oncolog$.tw. |
| 28 | myelodysplas$.tw. |
| 29 | chemotherapy.mp. |
| 30 | tumo?r$.mp. |
| 31 | carcinom$.mp. |
| 32 | or/24-31 |
| 33 | 17 and 23 |
| 34 | 32 and 33 |
| 35 | randomized controlled trial.pt. |
| 36 | controlled clinical trial.pt. |
| 37 | randomi?ed.ab. |
| 38 | placebo.ab. |
| 39 | drug therapy.fs. |
| 40 | randomly.ab. |
| 41 | trial.ab. |
| 42 | groups.ab. |
| 43 | or/35-42 |
| 44 | humans.sh. |
| 45 | 43 and 44 |
| 46 | 34 and 45 |
| 47 | limit 46 to ed=20080101-20081231 |
| 48 | randomized controlled trial.pt. |
| 49 | controlled clinical trial.pt. |
| 50 | randomized controlled trials as topic/ |
| 51 | random allocation/ |
| 52 | double blind method/ |
| 53 | single blind method/ |
| 54 | or/48-53 |
| 55 | (ANIMALS not HUMANS).sh. |
| 56 | 54 not 55 |
| 57 | clinical trial.pt. |
| 58 | exp clinical trial as topic/ |
| 59 | (clin$ adj25 trial$).ti,ab. |
| 60 | ((singl$ or doubl$ or trebl$ or tripl$) adj25 (blind$ or mask$)).ti,ab. |
| 61 | placebos/ |
| 62 | placebo$.ti,ab. |
| 63 | random$.ti,ab. |
| 64 | research design/ |
| 65 | or/57-64 |
| 66 | 65 not 55 |
| 67 | 66 not 56 |
| 68 | comparative study/ |
| 69 | exp evaluation studies/ |
| 70 | follow up studies/ |
| 71 | prospective studies/ |
| 72 | (control$ or prospectiv$ or volunteer$).ti,ab. |
| 73 | or/68-72 |
| 74 | 73 not 55 |
| 75 | 74 not (56 or 67) |
| 76 | 56 or 67 or 75 |
| 77 | 34 and 76 |
| 78 | 46 or 77 |
