# Supplementary material for: Does access to clinical study reports from the European Medicines Agency reduce reporting biases? A systematic review and meta-analysis of randomized controlled trials on the effect of erythropoiesis-stimulating agents in cancer patients
Source: PLoS One. 2017 Dec 11;12(12):e0189309. doi: 10.1371/journal.pone.0189309 (PMC5724886; doi:10.1371/journal.pone.0189309)
Supplement: S1 Study protocol — (DOCX) [file pone.0189309.s007.docx]

**Title: Does access to clinical study reports from the European Medicines Agency reduce reporting bias?**

**Protocol**

**Authors:**

Thomy Tonia^1^

Eliane Rohner^1^

Michael Grabik^1^

Frank Petavy^2^

Francesco Pignatti^2^

Peter Jüni^1^

Julia Bohlius^1^

**Affiliations:**

^1^ Institute of social and Preventive Medicine, University of Bern, Switzerland

^2^ European Medicines Agency, London, UK

*Background*

Randomised controlled trials and subsequent meta-analyses have shown that erythropoiesis stimulating agents (ESAs) reduce the risk of red blood cell transfusions at the cost of an increased risk of thrombo-vascular events (TVEs).^3-6^ While there is conflicting evidence on the effects of ESAs on overall survival, an individual patient data (IPD) meta-analysis showed increased mortality during the active study phase in cancer patients receiving ESAs compared to placebo or standard treatment.^1;2^ The effects of ESAs on tumour control and Quality of Life (QoL) are controversial.^6-13^

In view of safety concerns, the CHMP of the EMA recommends that “in cancer patients with a reasonably long life-expectancy, the benefit of using epoetins does not outweigh the risk of tumour progression and shorter overall survival and therefore the Committee concluded that in these patients anaemia should be corrected with blood transfusions”.^14^ The FDA has determined that ESAs are “not indicated for patients receiving myelosuppressive therapy when the anticipated outcome is cure”.^15^ Finally, guidelines of the American Society of Clinical Oncology and the American Society of Hematology recommend the use of ESAs as a treatment option in cancer patients receiving chemotherapy, with a haemoglobin level of less than 10 g/dL, and with the target of increasing haemoglobin to no more than needed to avoid transfusion.^16;17^

We recently updated^18^ our previous literature based review^3;4^ on the effects of ESAs in patients with cancer, integrating the evidence from the IPD meta-analysis published in 2009^1;2^ and recently published studies.^19-25^ However, of the 91 studies included in the meta-analysis none could be included in all outcomes assessed. Funnel plot analyses revealed that several outcomes showed asymmetric distributions, suggesting that beneficial but also harmful outcome events were over reported (see Table 1). Data were corrected using standard trim and fill methods.^26^ The corrected results did not change for short- and long-term mortality. For these two outcomes we had previously conducted analyses based on individual patient data and this type of data might be less affected by publication and outcome reporting biases. For the other outcomes assessed we used only data as reported in the published literature. In these outcomes after applying the trim and fill method the effect estimate was reduced by on average 18%, ranging between 3.6% for hypertension and 54% for Quality of Life, measured by FACT-Fatigue.

We conclude that outcome reporting bias is likely to have affected the results of our meta-analysis and would like to remedy this situation by retrieving additional outcome data from clinical study reports and other study related documents.

This study is hence intended to assess whether access to clinical study information available at EMA may help to reduce publication and outcome reporting biases.

2. Objective of the study

We aim to conduct a systematic review and meta-analysis on the effects of erythropoiesis-stimulating agents (ESAs) in cancer patients with anaemia or at risk of developing anaemia including reported and unreported as well as published and unpublished study results. We will investigate to which extend access to clinical study reports (CSR) held at the EMA is helping to reduce publication and outcome reporting biases in systematic reviews and meta-analyses.

Among the different activities to be undertaken to conduct the abovementioned review and meta-analysis, we will, inter alia:

1. identify all randomised controlled trials (RCTs) on the effects of ESAs in cancer patients;
2. use the CSR supplied by the EMA to identify outcome data which were not at all or not completely reported in the published literature or which were not published at all;
3. compare the data as reported in the literature with those reported in the CSR and investigate the impact of unreported and unpublished results on the overall effect estimates for ESAs in cancer patients;
4. compare the outcome data for short-term and long-term mortality between the CSRs, the published literature and the results from a previously conducted individual patient data meta-analysis on short- and long-term mortality;^1;2^

3. Methodology

This research protocol will define the methods used for study identification, quality assessment, data extraction and analyses.

1. *Study eligibility and identification*

We will identify all randomised controlled trials (RCTs) comparing epoetin or darbepoetin or any other erythropoiesis-stimulating agent versus placebo or standard treatment in cancer patients receiving or not receiving anti-cancer treatment, and who are anaemic or at risk of developing anaemia. Apart from the study drug, experimental and control arms have to receive identical treatment, with the exception of iron supplementation that can be administered differently in the arms of a given study. We will exclude studies if they were not truly randomised or had inadequately concealed treatment allocation, e.g. if participants were assigned to treatments in alternate order, or according to their birth dates, or the day of the week they arrived at the treatment centre. Studies with less than ten patients per study arm, as well as ongoing trials will be excluded. We will exclude trials on high-dose myeloablative chemotherapy regimens followed by bone marrow or peripheral blood stem cell transplantation, as well as trials using erythropoietin for short-term preoperative treatment to correct anaemia or to support collection of autologous blood prior to cancer surgery.

We will identify randomised controlled trials by conducting systematic searches in MEDLINE, EMBASE, the Cochrane Library, conference proceedings, reference lists of relevant systematic reviews^1-3;18;27^; and documents from Oncology Drug Advisory Committee (ODAC) hearings on the safety of ESAs.

For an example search strategy see **Appendix I**. Apart from the above databases, we will also search clinical trials registries (i.e. [www.ClinicalTrials.gov](http://www.ClinicalTrials.gov); http://apps.who.int/trialsearch; EudraCT; and ClinicalStudyResults.org) and contact experts in the field to identify studies we might have missed.

With these searches, we expect to identify approximately 100 eligible studies.

1. *Study documents*

For each eligible study we will retrieve

1. Publicly available information, i.e. full text publications, conference proceedings, secondary reports such as previous meta-analyses and ODAC documents. These documents will be referred to as “published literature”.
2. Clinical study reports and any related documents, i.e. clinical study reports including all appendices and annexes, study protocols including all appendices and annexes, as well as protocol amendments, and a blank case report form. (“RCT documentation”) and will be provided by the EMA.

We will assess and document for how many of the studies identified through literature searches (see above) RCT documentation is available at the EMA and if this RCT documentation is complete. We will also assess and document if there are any RCTs identified at EMA which had not been identified through the literature searches as outlined above.

1. *Data extraction*

We will extract data from published literature and the RCT documentation. For data extractions we will use a standardised data extraction form. This form will include the following items:

- General information: title, authors, source, contact address of corresponding author, year of publication, any duplicate publications, trial setting, recruitment dates, funding.
- Trial characteristics: design, method of randomisation, concealment of allocation, blinding of patients and clinicians.
- Patients: sampling, inclusion and exclusion criteria, sample size, baseline characteristics, similarity of groups at baseline, diagnostic criteria, withdrawals, losses to follow-up.
- Interventions: placebo use, dose, dosing regimen, duration, route of administration, red blood cell transfusion trigger, co-medications with dose, route and timing.
- Outcomes: outcomes as specified below.

1. *Outcomes*

We will assess the following outcomes:

- Short-term mortality, typically defined as mortality during the active study period plus 30 days follow up;
- Long-term mortality, defined as mortality during the longest follow-up available;
- Patients receiving red blood cell transfusions and number of red blood cell units transfused;
- Haemoglobin change and haematological response, typically defined as haemoglobin increase of 2 g/dL independent from red blood cell transfusions;
- Tumour control and disease progression;
- Quality of Life, measured with FACT-F, FACT-An and other standardized instruments;
- Adverse events, including thromboembolic events, hypertension, haemorrhage/ thrombocytopenia, rash/irritation/pruritus, seizures and other.

1. *Quality assessment*

For quality assessment two review authors of the analysis team will independently assess the full text articles and the RCT documentation of the eligible studies. Disagreements arising at any stage will be resolved by discussion and consensus.

We will use generally accepted criteria, such as method of randomisation, concealment of allocation, blinding of study participants, study clinicians and outcome assessors, as well as drop outs and losses to follow up and adherence to intention-to-treat (ITT) principle.

For specific outcomes, such as tumour control and Quality of Life, additional criteria will be considered. We will test the effect of individual quality variables (e.g. allocation, blinding, ITT) on the estimated effect of ESAs in subgroup analyses. We will not use summary scores of quality parameter because of their previously reported shortcomings.^28^

We will specifically compare the quality of reporting in the published literature and the RCT documentation.

1. *Statistical analyses*

We will perform meta-analyses according to the recommendations of the Cochrane Handbook for Systematic Reviews of Interventions.^29^

We will assume a random-effects model for all meta-analyses. Fixed effects meta-analyses will be conducted to test whether results are robust. For binary data, we will use the risk ratio as a measure of treatment effect and we will use the inverse variance method for pooling. For continuous data, we will calculate the mean differences if the outcome was measured on the same scale in all trials. For time to event data we will calculate the hazard ratio using methods described in Parmar et al^30^ or binary mortality data.

We will use P values of the homogeneity test to assess heterogeneity across trials. Potential causes of heterogeneity will be explored by predefined subgroup and sensitivity analyses. In addition to subgroup analyses we will conduct random-effects meta-regression (see section 9.6.4, Cochrane Handbook^29^) for outcomes with evidence of statistical heterogeneity. We will use the DerSimonian-Laird method to estimate the between study variance in meta-regression. For model selection, we will consider all covariates showing a significant effect (P < 0.05) in a univariate analysis. We will restrict the analysis to studies providing information on all variables that are statistically significant in univariate analyses.

We will compare outcome results for a given study reported in different sources, i.e. clinical study reports, publications and secondary sources such as meta-analyses, ODAC documents and short reports listed in clinical trials data bases. Results for one study stemming from different sources will be compared for consistency. Discrepant results will be noted and reasons for discrepancies investigated and reported. In meta-analyses with at least ten trials, we will assess small study bias with funnel plots and linear regression tests.^31^ We will use trim-and-fill^26^ and Copas selection model^32^ to adjust for potential publication bias in meta-analyses based on 1) the published data only and 2) based on published and unpublished data.

In general we will conduct subgroup analyses for relevant patient, treatment and study design related characteristics, i.e. haemoglobin level at study entry, target haemoglobin, tumour type, age, type of anticancer treatment given, type of ESA given, duration of ESA medication, iron supplementation, source of data (published data versus data from clinical study reports) and study quality parameters (concealment of allocation, masking, intention-to-treat analysis, other).

Analyses will be conducted in 1) all RCTs regardless of anticancer treatment and 2) in RCTs using chemotherapy. For the analysis of patient related outcome measures, such as QoL, we will conduct additional analyses restricted to placebo-controlled studies.

We will perform all analyses using Review Manager (RevMan) 5.1 and STATA 12 software packages.

**Table 1: Re-analysis of literature based Cochrane Review^18^ on the effects of ESAs in cancer patients before and after trim and fill^26^ correction**

|  | **Studies included** | **P-value*** | **Data as reported^18^** | **After trim and fill^26^** | **Percentage change** |
| --- | --- | --- | --- | --- | --- |
|  |  |  | **ESA versus control** (95% CI) | **ESA versus control** (95% CI) |  |
| Short-term mortality | 78 | 0.6931 | HR 1.17 (1.06-1.29) | HR 1.17 (1.06-1.29) | 0% |
| Long-term mortality | 78 | 0.9209 | HR 1.05 (0.98-1.12) | HR 1.05 (0.98-1.12) | 0% |
| Hypertension | 31 | <0.001 | RR 1.12 (0.94-1.34) | RR 1.08 (0.86-1.35) | 3.6% |
| Thrombo-vascular events | 57 | 0.01455 | RR 1.47 (1.29-1.68) | RR 1.39 (1.22-1.59) | 5.4% |
| Number of red blood cell units transfused | 19 | 0.5484 | MD -0.99 (-1.30- -0.68) | MD -0.93 (-1.24,-0.62) | 6.1% |
| Haemoglobin change | 56 | 0.5993 | MD 1.60 (1.41-1.80) | MD 1.32 (1.11-1.54) | 17.5% |
| Haematologic response | 33 | <0.001 | RR 3.45 (2.91-4.10) | RR 2.75 (2.30-3.30) | 20.3% |
| Red blood cell transfusions | 70 | <0.0001 | RR 0.59 (0.54-0.64) | RR 0.72 (0.66-0.70) | 22.0% |
| FACT-Fatigue | 18 | 0.02772 | MD 2.37 (1.40-3.35) | MD 1.09 (-0.01-2.19) | 54% |

Results are sorted by percentage change (last column). ESAs erythropoiesis-stimulating agents, HR hazard ratio, RR relative risk, MD mean difference, CI confidence interval. Meta-analyses done with random-effects models. *Linear regression test for funnel plot asymmetry

**Reference List**

(1) Bohlius J, Schmidlin K, Brillant C, Schwarzer G, Trelle S, Seidenfeld J et al. Recombinant human erythropoiesis-stimulating agents and mortality in patients with cancer: a meta-analysis of randomised trials. *Lancet* 2009; 373(9674):1532-1542.

(2) Bohlius J, Schmidlin K, Brillant C, Schwarzer G, Trelle S, Seidenfeld J et al. Erythropoietin or Darbepoetin for patients with cancer--meta-analysis based on individual patient data. *Cochrane Database Syst Rev* 2009;(3):CD007303.

(3) Bohlius J, Wilson J, Seidenfeld J, Piper M, Schwarzer G, Sandercock J et al. Erythropoietin or darbepoetin for patients with cancer. *Cochrane Database Syst Rev* 2006; 3:CD003407.

(4) Bohlius J, Wilson J, Seidenfeld J, Piper M, Schwarzer G, Sandercock J et al. Recombinant human erythropoietins and cancer patients: updated meta-analysis of 57 studies including 9353 patients. *J Natl Cancer Inst* 2006; 98(10):708-714.

(5) Seidenfeld J, Piper M, Bohlius J, Weingart O, Trelle S, Engert A et al. Comparative effectiveness of epoetin and darbepoetin for managing anemia in patients undergoing cancer treatment. Agency for Healthcare Research and Quality, editor. 2006. Rockville.

(6) Tonelli M, Hemmelgarn B, Reiman T, Manns B, Reaume MN, Lloyd A et al. Benefits and harms of erythropoiesis-stimulating agents for anemia related to cancer: a meta-analysis. *CMAJ* 2009; 180(11):E62-E71.

(7) Ross SD, Allen IE, Henry DH, Seaman C, Sercus B, Goodnough LT. Clinical benefits and risks associated with epoetin and darbepoetin in patients with chemotherapy-induced anemia: a systematic review of the literature. *Clin Ther* 2006; 28(6):801-831.

(8) Minton O, Richardson A, Sharpe M, Hotopf M, Stone P. A systematic review and meta-analysis of the pharmacological treatment of cancer-related fatigue. *J Natl Cancer Inst* 2008; 20;100(16):1155-1166.

(9) Minton O, Richardson A, Sharpe M, Hotopf M, Stone P. Drug therapy for the management of cancer-related fatigue. *Cochrane Database Syst Rev* 2010;(7):CD006704.

(10) Wilson J, Yao GL, Raftery J, Bohlius J, Brunskill S, Sandercock J et al. A systematic review and economic evaluation of epoetin alpha, epoetin beta and darbepoetin alpha in anaemia associated with cancer, especially that attributable to cancer treatment. *Health Technol Assess* 2007; 11(13):1-iv.

(11) Cella D, Kallich J, McDermott A, Xu X. The longitudinal relationship of hemoglobin, fatigue and quality of life in anemic cancer patients: results from five randomized clinical trials. *Ann Oncol* 2004; 15(6):979-986.

(12) Jones M, Schenkel B, Just J, Fallowfield L. Epoetin alfa improves quality of life in patients with cancer: results of metaanalysis. *Cancer* 2004; 101(8):1720-1732.

(13) Kimel M, Leidy NK, Mannix S, Dixon J. Does epoetin alfa improve health-related quality of life in chronically ill patients with anemia? Summary of trials of cancer, HIV/AIDS, and chronic kidney disease. *Value Health* 2008; 11(1):57-75.

(14) Press release: EMEA recommends a new warning for epoetins for their use in cancer patients. http://www.emea.europa.eu/docs/en_GB/document_library/Press_release/2009/11/WC500015069.pdf . 26-6-2008.

(15) FDA Drug Safety Communication: Erythropoiesis-Stimulating Agents (ESAs): Procrit, Epogen and Aranesp. http://www.fda.gov/Drugs/DrugSafety/PostmarketDrugSafetyInformationforPatientsandProviders/ucm200297.htm . 26-2-2010.

(16) Rizzo JD, Brouwers M, Hurley P, Seidenfeld J, Arcasoy MO, Spivak JL et al. American Society of Clinical Oncology/American Society of Hematology clinical practice guideline update on the use of epoetin and darbepoetin in adult patients with cancer. *J Clin Oncol* 2010; 20;28(33):4996-5010.

(17) Rizzo JD, Brouwers M, Hurley P, Seidenfeld J, Arcasoy MO, Spivak JL et al. American Society of Hematology/American Society of Clinical Oncology clinical practice guideline update on the use of epoetin and darbepoetin in adult patients with cancer. *Blood* 2010; 116(20):4045-4059.

(18) Tonia T, Mettler A, Robert N, Schwarzer G, Seidenfeld J, Weingart O et al. Erythropoietin or darbepoetin for patients with cancer. *Cochrane Database Syst Rev* 2012; 12:CD003407.

(19) Gupta S, Singh PK, Bisth SS, Bhatt ML, Pant M, Gupta R et al. Role of recombinant human erythropoietin in patients of advanced cervical cancer treated "by chemoradiotherapy". *Cancer Biol Ther* 2009; 8(1):13-17.

(20) Engert A, Josting A, Haverkamp H, Villalobos M, Lohri A, Sokler M et al. Epoetin alfa in patients with advanced-stage Hodgkin's lymphoma: results of the randomized placebo-controlled GHSG HD15EPO trial. *J Clin Oncol* 2010; 28(13):2239-2245.

(21) Buchner A, Bias P. Epoetin theta shows efficacy and safety in a placebo controlled, randomized phase III study in cancer patients receiving non-platinum chemotherapy. Onkologie 32, suppl. 4[88]. 2009.

(22) Krzakowski M. Epoetin delta: efficacy in the treatment of anaemia in cancer patients receiving chemotherapy. *Clin Oncol (R Coll Radiol )* 2008; 20(9):705-713.

(23) Randomized study of darbepoetin alfa as modifier of radiotherapy in patients with primary squamous cell carcinoma of the head and neck (HNSCC): Final outcome of the DAHANCA 10 trial. Annual Meeting of the American Society of Clinical Oncology; 2009.

(24) Tsuboi M, Ezaki K, Tobinai K, Ohashi Y, Saijo N. Weekly administration of epoetin beta for chemotherapy-induced anemia in cancer patients: results of a multicenter, Phase III, randomized, double-blind, placebo-controlled study. *Jpn J Clin Oncol* 2009; 39(3):163-168.

(25) Tjulandin SA, Bias P, Elsasser R, Gertz B, Kohler E, Buchner A. Epoetin Theta in Anaemic Cancer Patients Receiving Platinum-Based Chemotherapy: A Randomised Controlled Trial. *Arch Drug Inf* 2010; 3(3):45-53.

(26) Carpenter JR, Schwarzer G, Rucker G, Kunstler R. Empirical evaluation showed that the Copas selection model provided a useful summary in 80% of meta-analyses. *J Clin Epidemiol* 2009; 62(6):624-631.

(27) Bohlius J, Langensiepen S, Schwarzer G, Seidenfeld J, Piper M, Bennett C et al. Recombinant human erythropoietin and overall survival in cancer patients: results of a comprehensive meta-analysis. *J Natl Cancer Inst* 2005; 97(7):489-498.

(28) Juni P, Witschi A, Bloch R, Egger M. The hazards of scoring the quality of clinical trials for meta-analysis. *JAMA* 1999; 282(11):1054-1060.

(29) Cochrane Handbook for Systematic Reviews of Interventions Version 5.1.0 [updated March 2011]. The Cochrane Collaboration; 2011.

(30) Parmar MK, Torri V, Stewart L. Extracting summary statistics to perform meta-analyses of the published literature for survival endpoints. *Stat Med* 1998; 17(24):2815-2834.

(31) Egger M, Davey SG, Schneider M, Minder C. Bias in meta-analysis detected by a simple, graphical test. *BMJ* 1997; 315(7109):629-634.

(32) Duval S. The trim and fill method. Publication bias in meta-analysis - Prevention, assessment and adjustments. Chichester: Wiley; 2005. 127-144.

Appendix 1

Search strategy to be used in EMBASE

**01. 'erythropoietin'** OR **'recombinant erythropoietin'**/exp

**02. eryt*ropo***

**03 eritropo***

**04. epo***

**05. hematopo*etin***

**06. hemopo*etin***

**07. haematopo*etin***

**08. haemopo*etin***

**09. eprex***

**10. neorecormon***

**11. aranesp***

**12. procit***

**13. cepo***

**14. darbepo?eti***

**15. cera***

16. **continuous** AND **erythropo?es*** AND (**'receptor'**/exp OR **receptor**) AND **activator**

**17. methoxy** AND **polyethylene** AND **'glycol epoetin'** AND **beta**

**18. mircer***

**19. micer***

**20. 'erythropoietin receptor'**/exp OR **'erythropoietin receptor'**

**21. #1** OR **#2** OR **#3** OR **#4** OR **#5** OR **#6** OR **#7** OR **#8** OR **#9** OR **#10** OR **#11** OR **#12** OR **#13** OR **#14** OR **#15** OR **#16** OR **#17** OR **#18** OR **#19** OR **#20**

**22. 'anemia'**/exp OR **anemia**

**23. anaemia**

**24. anemia**

**25. anaemi***

**26. anemi***

**27. #22** OR **#23** OR **#24** OR **#25** OR **#26**

**28. 'neoplasm'**/exp OR **neoplasm**

**29. malignan***

**30. cancer***

**31. oncolog***

**32. myelodysplas***

**33. chemotherapy**

**34. tumo?r***

**35. carcinoma***

**36. #28** OR **#29** OR **#30** OR **#31** OR **#32** OR **#33** OR **#34** OR **#35**

**37. #21** AND **#27**

**38. #36** AND **#37**

**39. 'clinical trial'**/exp OR **'clinical trial'**

**40. 'randomized controlled trial'**/exp OR **'randomized controlled trial'**

**41. 'randomization'**/exp OR **'randomization'**

**42. 'single blind procedure'**/exp OR **'single blind procedure'**

**43. 'double blind procedure'**/exp OR **'double blind procedure'**

**44. 'crossover procedure'**/exp OR **'crossover procedure'**

**45. 'placebo'**/exp OR **placebo**

**46. randomi?ed** AND **controlled** AND **trial?**

**47. rct**

**48. 'random allocation'**

**49. 'randomly allocated'**

**50. 'allocated randomly'**

**51. 'allocated'** NEAR/2 **'random'**

**52. single** AND **blind***

**53. double** AND **blind***

**54. 'treble'** NEAR/5 **'blind'**

**55. 'triple'** NEAR/5 **'blind'**

**56. placebo***

**57. 'prospective study'**

**58. #39** OR **#40** OR **#41** OR **#42** OR **#43** OR **#44** OR **#45** OR **#46** OR **#47** OR **#48** OR **#49** OR **#50** OR **#51** OR **#52** OR **#53** OR **#54** OR **#55** OR **#56** OR **#57**

**59. 'case study'**

**60. 'case report'**

**61. 'abstract report'** OR **letter**

**62. #59** OR **#60** OR **#61**

**63. #58** NOT **#62**

**64. 'animal'**/exp OR **animal**

**65. 'human'**/exp OR **human**

**66. #64** NOT **#65**

**67. #63** NOT **#66**

**68. #38** AND **#67**

**69. #68** AND [1-8-2009]/sd NOT [11-1-2011]/sd
