# Supplementary material for: Does access to clinical study reports from the European Medicines Agency reduce reporting biases? A systematic review and meta-analysis of randomized controlled trials on the effect of erythropoiesis-stimulating agents in cancer patients
Source: PLoS One. 2017 Dec 11;12(12):e0189309. doi: 10.1371/journal.pone.0189309 (PMC5724886; doi:10.1371/journal.pone.0189309)
Supplement: S1 Text — (DOCX) [file pone.0189309.s008.docx]

**S1 Text: Protocol changes**

The following outcomes had been planned at protocol stage but were disregarded in the analyses because of limited resources: tumor control and disease progression; hemorrhage/ thrombocytopenia, rash/irritation/pruritus, seizures. In post-hoc sensitivity analyses, we merged all experimental arms into a single arm for studies with more than one experimental arm. A full exploration of heterogeneity between trials will be presented in the update of the Cochrane Review [1].

**References**

1. Tonia T, Mettler A, Robert N, Schwarzer G, Seidenfeld J, Weingart O, et al. Erythropoietin or darbepoetin for patients with cancer. Cochrane database Syst Rev. 2012;12: CD003407.
