# Supplementary material for: Does access to clinical study reports from the European Medicines Agency reduce reporting biases? A systematic review and meta-analysis of randomized controlled trials on the effect of erythropoiesis-stimulating agents in cancer patients
Source: PLoS One. 2017 Dec 11;12(12):e0189309. doi: 10.1371/journal.pone.0189309 (PMC5724886; doi:10.1371/journal.pone.0189309)
Supplement: S2 Text — (DOCX) [file pone.0189309.s009.docx]

**S2 Text: Handling of incompletely reported outcome data**

If outcome data were incompletely reported the following rules applied. If size of group for a given outcome was not reported, we used the number of patients randomized into the group. If standard deviations (SD) were not reported, we calculated them using the reported standard errors (SE) or 95% confidence intervals (CI). If SDs, SEs, or CIs were not reported, we used the reported range and divided it by four to approximate the SD. If none of these was reported, we imputed the SD by pooling the SDs from other studies using both SDs reported in the public domain and in the EMA documentation. If data for a given outcome were reported in several sources, all data were extracted and the data that were most completely reported with population size closest to the ITT population were chosen for analysis. We excluded data that were reported only qualitatively.
