# Supplementary material for: Does access to clinical study reports from the European Medicines Agency reduce reporting biases? A systematic review and meta-analysis of randomized controlled trials on the effect of erythropoiesis-stimulating agents in cancer patients
Source: PLoS One. 2017 Dec 11;12(12):e0189309. doi: 10.1371/journal.pone.0189309 (PMC5724886; doi:10.1371/journal.pone.0189309)
Supplement: S1 Table — CLL, chronic lymphocytic leukemia; MM, multiple myeloma; NHL, non-Hodgkin lymphoma; ODAC, Oncologic Drugs Advisory Committee; pc, personal communication; Q2W, every second week; Q3W, every third week; Q4W, every fourth week; sc, subcutaneous; TIW, three times per week. (DOCX) [file pone.0189309.s010.docx]

**S1 Table: Characteristics of eligible trials identified at the European Medicines Agency (EMA)**

| **Protocol Name** | **Study author** | **Public domain data sources** | **Study type** | Allocation randomized | Allocation concealed | Drug and Dose | **Number of participants (ITT)**  **ESA/Control** | **Cancer details** | **Recruitment of patients** |
| --- | --- | --- | --- | --- | --- | --- | --- | --- | --- |
| **Roche/Boehringer Mannheim (Roche since 1997)** | | | | | | | | | |
| Protocol BA 16756 (BRAVE)  Research Report 1017160 | Aapro 2008^1^ | Full text, IPD Review^2,3^ | Phase III | Unclear | Yes | Epoetin beta vs standard care  30000 IU sc weekly | 231/232 | Metastatic breast cancer | Nov 2002 – Jun 2004 |
| Protocol MF 4467  Research Report B-171622 | Osterborg 2002^4^ | Full text, pc published in ^5^, IPD Review^2,3^ | Phase III | Yes | Yes | Epoetin beta vs placebo  150 IU/kg sc TIW | 173/176 | MM, NHL, CLL | Jun 1997 – Dec 1999 |
| Protocol MF 4421  Research Report B-172512 | Boogaerts 2003^6^ | Full text, pc published in ^5^, IPD Review^2,3^ | Phase IIIb | Unclear | Yes | Epoetin beta vs standard care  150 IU/kg sc TIW | 133/129 | MM, NHL, CLL, ovarian cancer and other solid tumors | Oct 1996 –Sept 1998 |
| Study No.: MF 4321  Report No.: O 17 | Oberhoff 1998^7^ | Full text, pc published in ^5^, IPD Review^2,3^ | Phase II/III | Unclear | Yes | Epoetin beta vs standard care  7 x 5000 IU sc weekly | 117/110 | Gynecological cancer, lung cancer, breast cancer and other solid tumors | Apr 1992 – Sept 1994 |
| Study No.: MF 4250  Report No.: O 11 | Osterborg 1996^8^ | Full text, pc published in ^5^, IPD Review^2,3^ | Phase II/III | Unclear | Yes | Epoetin beta vs standard care  a: 7 x 10000 IU/kg sc weekly  b: titration | 99/49 | MM, NHL | Jun 1992 – May 1993 |
| Study No.: MF 4313  Report No.: O 13 | Cazzola 1995^9^ | Full text, pc published in ^5^, IPD Review^2,3^ | Phase II | Unclear | Unclear | Epoetin beta vs standard care  a: 7 x 1000 IU sc weekly  b: 7 x 2000 IU sc weekly  c: 7 x 5000 IU sc weekly  d: 7 x 10000 IU sc weekly | 117/29 | MM, NHL | Mar 1992 – Jun 1993 |
| Study No.: MF 4249  Report No.: O 14 | Ten Bokkel 1998^10^ | Full text, pc published in ^5^, IPD Review^2,3^ | Phase III | Unclear | Yes | Epoetin beta vs standard care  a: 150 IU/kg sc TIW  b: 300 IU/kg sc TIW | 88/34 | Ovarian cancer | Sep 1991 – Mar 1994 |
| Protocol MO 16375  Research Report 1005876 | Strauss 2008^11^ | Full text, IPD Review^2,3^ | Phase IV | Unclear | Yes | Epoetin beta vs standard care  150 IU/kg sc TIW | 34/40 | Cervical cancer | Oct 2001 – Oct 2003 |
| Protocol MF4449  Research Report 1009765 | Henke 2003^12^ | Full text, ODAC documents, IPD Review^2,3^ | Phase II | Unclear | Yes | Epoetin beta vs placebo  300 IU/kg sc TIW | 180/171 | Squamous cell carcinoma of the oral cavity, oropharynx, hypopharynx, or larynx | Mar 1997 – Nov 2002 |
| Protocol MF4362  Research Report 1022737 | Unpublished^13^ | None | Phase III | Unclear | Unclear | Epoetin beta vs standard care  150 U/kg sc TIW maximum of 9 months | 12/12 | Ewing’s sarcoma, osteosarcoma, rhabdomyosarcoma and other solid tumors | Feb 1994 – Sep 1997 |
| **Amgen** |  |  |  |  |  |  |  |  |  |
| Study No.: 20010145  IND No.: 8223 | Pirker 2008^14^ | Full text, IPD Review^2,3^ | Phase III | Unclear | Yes | Darbepoetin alpha vs placebo  300 µg sc weekly for weeks 1-4 then 300 µg Q3W starting week 5 onwards | 299/301 | Extensive-stage small cell lung cancer | Dec 2002 – unclear (cutoff Feb 2007) |
| Study No.: 20010103  EMEA/H/C/332 and EMEA/H/C/333 | Smith 2008^15^ | Full text, IPD Review^2,3^ | Phase III | Unclear | Yes | Darbepoetin alpha vs placebo  6.75 µg/kg sc Q4W | 517/472 | Non-small cell lung cancer, breast cancer, prostate cancer and other non-myeloid malignancies | Apr 2004 – unclear (cutoff Nov 2006) |
| PREPARE – GBG 49 | Untch 2011^16^ | Full text, IPD Review^2,3^ | Phase III | Unclear | Unclear | Darbpoetin alpha vs standard care  4.5 µg/kg sc Q2W until 14 days after last chemotherapy | 356/377 | Non-metastatic breast cancer | Jun 2002 – unclear (cutoff Oct 2008) |
| Study No.: DE-2002-0015 (AraPlus)  Protocol No.: WSG AM-03 | Nitz 2014^17^ | Full text | Phase III | Yes | Yes | Darbepoetin alpha vs standard care  DA 300 μg sc Q3W | 619/615 | Non-metastatic breast cancer | Jan 2004 – Jun 2008 |
| **BioGeneriX AG** |  |  |  |  |  |  |  |  |  |
| BioGeneriX Study No.: XM01-21  EudraCT No.: 2005-001419-23 | Tjulandin 2010^18^ | Full text | Phase III | Yes | Yes | Epoetin theta and epoetin beta vs placebo  a: 20000 IU epoetin theta sc weekly  b: 150 IU/kg sc epoetin beta TIW | 149/74 | Ovarian epithelial cancer, gastric cancer, squamous cell lung carcinoma, breast cancer and other solid tumors | Oct 2005 – Apr 2007 |
| BioGeneriX Study No.: CSR XM01-22  EudraCT No.: 2005-001421-28 | Tjulandin 2011^19^ | Full text | Phase III | Yes | Yes | Epoetin theta vs placebo  20000 IU sc weekly | 95/91 | MM, breast cancer, CLL, gastric cancer and other solid tumors or non-myeloid hemato-logical tumors | Nov 2005 – Feb 2007 |
| BioGeneriX Study No.: CSR XM01-23  EudraCT No.: 2005-001422-89 | Unpublished^20^ | none | Phase III | Yes | Yes | Epoetin theta vs placebo  20000 – 60000 IU sc weekly | 90/87 | MM, NHL, CLL | Jul 2005 – Dec 2007 |

CLL, chronic lymphocytic leukemia; MM, multiple myeloma; NHL, non-Hodgkin lymphoma; ODAC, Oncologic Drugs Advisory Committee; pc, personal communication; Q2W, every second week; Q3W, every third week; Q4W, every fourth week; sc, subcutaneous; TIW, three times per week.

**References**

(1) Aapro M, Leonard RC, Barnadas A et al. Effect of once-weekly epoetin beta on survival in patients with metastatic breast cancer receiving anthracycline- and/or taxane-based chemotherapy: results of the Breast Cancer-Anemia and the Value of Erythropoietin (BRAVE) study. *J Clin Oncol* 2008;26:592-598.

(2) Bohlius J, Schmidlin K, Brillant C et al. Erythropoietin or Darbepoetin for patients with cancer--meta-analysis based on individual patient data. *Cochrane Database Syst Rev* 2009;CD007303.

(3) Bohlius J, Schmidlin K, Brillant C et al. Recombinant human erythropoiesis-stimulating agents and mortality in patients with cancer: a meta-analysis of randomised trials. *Lancet* 2009;373:1532-1542.

(4) Osterborg A, Brandberg Y, Molostova V et al. Randomized, double-blind, placebo-controlled trial of recombinant human erythropoietin, epoetin Beta, in hematologic malignancies. *J Clin Oncol* 2002;20:2486-2494.

(5) Bohlius J, Langensiepen S, Schwarzer G et al. Erythropoietin for patients with malignant disease. *The Cochrane Library* 2005;4.

(6) Boogaerts M, Coiffier B, Kainz C, and the Epoetin beta QOL Working Group. Impact of epoetin beta on quality of life in patients with malignant disease. *Br J Cancer* 2003;88:988-995.

(7) Oberhoff C, Neri B, Amadori D et al. Recombinant human erythropoietin in the treatment of chemotherapy- induced anemia and prevention of transfusion requirement associated with solid tumors: a randomized, controlled study. *Ann Oncol* 1998;9:255-60.

(8) Osterborg A, Boogaerts MA, Cimino R et al. Recombinant human erythropoietin in transfusion-dependent anemic patients with multiple myeloma and non-Hodgkin´s lymphoma - a randomized multicenter study. *Blood* 1996;87:2675-82.

(9) Cazzola M, Messinger D, Battistel V et al. Recombinant human erythropoietin in the anemia associated with multiple myeloma or non-Hodgkin´s lymphoma: dose finding and identification of predictors of response. *Blood* 1995;86:4446-53.

(10) Ten Bokkel Huinink WW, De Swart CA, Van Toorn DW et al. Controlled multicentre study of the influence of subcutaneous recombinant human erythropoietin on anaemia and transfusion dependency in patients with ovarian carcinoma treated with platinum-based chemotherapy. *Medical Oncology* 1998;15:174-82.

(11) Strauss HG, Haensgen G, Dunst J et al. Effects of anemia correction with epoetin beta in patients receiving radiochemotherapy for advanced cervical cancer. *Int J Gynecol Cancer* 2008;18:515-24.

(12) Henke M, Laszig R, Ruebe C et al. Erythropoietin to treat head and neck cancer patients with anaemia undergoing radiotherapy: randomised, double-blind, placebo-controlled trial. *Lancet* 2003;362:1255-60.

(13) Clinical Study Report MF4362 - Open-label, randomized phase III study to investigate the effect of epoetin beta on blood transfusion need in children with various malignant solid tumours treated with chemotherapy. Research Report - 1022737, August 16, 2006.

(14) Pirker R, Ramlau RA, Schuette W et al. Safety and efficacy of darbepoetin alpha in previously untreated extensive-stage small-cell lung cancer treated with platinum plus etoposide. *J Clin Oncol* 2008;26:2342-2349.

(15) Smith RE, Jr., Aapro MS, Ludwig H et al. Darbepoetin alpha for the treatment of anemia in patients with active cancer not receiving chemotherapy or radiotherapy: results of a phase III, multicenter, randomized, double-blind, placebo-controlled study. *J Clin Oncol* 2008;26:1040-1050.

(16) Untch M, von MG, Konecny GE et al. PREPARE trial: a randomized phase III trial comparing preoperative, dose-dense, dose-intensified chemotherapy with epirubicin, paclitaxel, and CMF versus a standard-dosed epirubicin-cyclophosphamide followed by paclitaxel with or without darbepoetin alfa in primary breast cancer--outcome on prognosis. *Ann Oncol* 2011;22:1999-2006.

(17) Nitz U, Gluz O, Zuna I et al. Final results from the prospective phase III WSG-ARA trial: impact of adjuvant darbepoetin alfa on event-free survival in early breast cancer. *Ann Oncol* 2014;25:75-80.

(18) Tjulandin SA, Bias P, Elsasser R, Gertz B, Kohler E, Buchner A. Epoetin Theta in Anaemic Cancer Patients Receiving Platinum-Based Chemotherapy: A Randomised Controlled Trial. *Arch Drug Inf* 2010;3:45-53.

(19) Tjulandin SA, Bias P, Elsasser R, Gertz B, Kohler E, Buchner A. Epoetin Theta with a New Dosing Schedule in Anaemic Cancer Patients Receiving Nonplatinum-Based Chemotherapy: A Randomised Controlled Trial. *Arch Drug Inf* 2011;4:33-41.

(20) Efficacy and safety of XM01 compared to placebo in anaemic patients with low grade non-Hodgkin's lymphoma, chronic lymphocytic leukaemia or multiple myeloma receiving anticancer therapy (CSR XM01-23). 2008.
