# Supplementary material for: Does access to clinical study reports from the European Medicines Agency reduce reporting biases? A systematic review and meta-analysis of randomized controlled trials on the effect of erythropoiesis-stimulating agents in cancer patients
Source: PLoS One. 2017 Dec 11;12(12):e0189309. doi: 10.1371/journal.pone.0189309 (PMC5724886; doi:10.1371/journal.pone.0189309)
Supplement: S2 Table — (DOCX) [file pone.0189309.s011.docx]

**S2 Table: Completeness of RCT documentation provided by the European Medicines Agency**

|  | Pirker 2008^1^ | XM01-23^2^ | Tjulandin 2010^3^ | Tjulandin 2011^4^ | Smith 2008 ^5^ | Oberhoff 1998^6^ | Osterborg 2002^7^ | Henke 2003^8^ | Aapro 2008^9^ | Osterborg 1996^10^ | Ten Bokkel 1998^11^ | Cazzola 1995^12^ | Nitz 2014^13^ | Strauss 2008^14^ | Boogaerts 2003^15^ | MF4362^16^ | Untch 2011^17^ |
| --- | --- | --- | --- | --- | --- | --- | --- | --- | --- | --- | --- | --- | --- | --- | --- | --- | --- |
| Synopsis | Yes | No | No | No | Yes | Yes | Yes | Yes | Yes | Yes | Yes | Yes | Yes | Yes | Yes | Yes | No |
| Efficacy evaluation | Yes | Yes | Yes | Yes | Yes | Yes | Yes | Yes | Yes | Yes | Yes | Yes | Yes | Yes | Yes | Yes | Yes |
| Safety evaluation | Yes | Yes | Yes | Yes | Yes | Yes | Yes | Yes | Yes | Yes | Yes | Yes | Yes | Yes | Yes | Yes | Yes |
| Study protocol | Yes | Yes | Yes | Yes | Yes | Yes | Yes | Yes | Yes | Yes | Yes | Yes | Yes | Yes | No | No | No |
| Blank case report forms | Yes | Yes | Yes | Yes | Yes | Yes | Yes | Yes | Yes | Yes | Yes | Yes | Yes | Yes | No | No | No |
| Completed case report forms | No | No | No | No | No | No | No | No | No | No | No | No | No | No | No | No | No |
| Statistical analysis plan | Yes | Yes | Yes | Yes | Yes | Yes | Yes | Yes | Yes | Yes | Yes | Yes | Yes | Yes | No | No | No |
| Individual participant listings* | Yes | Yes | Yes | Yes | No | Yes | Yes | Yes | Yes | No | No | No | No | Yes | No | Yes | No |
| Individual participant  listings** | Yes | Yes | Yes | Yes | Yes | Yes | Yes | Yes | Yes | Yes | Yes | Yes | Yes | Yes | Yes | Yes | No |
| Total number of pages | **14,569** | **6,321** | **5,703** | **5,618** | **4,692** | **2,973** | **2,613** | **2,137** | **1,825** | **1,370** | **1,105** | **947** | **940** | **808** | **425** | **169** | **72** |

* for selected efficacy outcomes

** for safety outcomes

**References**

(1) Pirker R, Ramlau RA, Schuette W et al. Safety and efficacy of darbepoetin alpha in previously untreated extensive-stage small-cell lung cancer treated with platinum plus etoposide. *J Clin Oncol* 2008;26:2342-2349.

(2) Efficacy and safety of XM01 compared to placebo in anaemic patients with low grade non-Hodgkin's lymphoma, chronic lymphocytic leukaemia or multiple myeloma receiving anticancer therapy (CSR XM01-23). 2008.

(3) Tjulandin SA, Bias P, Elsasser R, Gertz B, Kohler E, Buchner A. Epoetin Theta in Anaemic Cancer Patients Receiving Platinum-Based Chemotherapy: A Randomised Controlled Trial. *Arch Drug Inf* 2010;3:45-53.

(4) Tjulandin SA, Bias P, Elsasser R, Gertz B, Kohler E, Buchner A. Epoetin Theta with a New Dosing Schedule in Anaemic Cancer Patients Receiving Nonplatinum-Based Chemotherapy: A Randomised Controlled Trial. *Arch Drug Inf* 2011;4:33-41.

(5) Smith RE, Jr., Aapro MS, Ludwig H et al. Darbepoetin alpha for the treatment of anemia in patients with active cancer not receiving chemotherapy or radiotherapy: results of a phase III, multicenter, randomized, double-blind, placebo-controlled study. *J Clin Oncol* 2008;26:1040-1050.

(6) Oberhoff C, Neri B, Amadori D et al. Recombinant human erythropoietin in the treatment of chemotherapy- induced anemia and prevention of transfusion requirement associated with solid tumors: a randomized, controlled study. *Ann Oncol* 1998;9:255-60.

(7) Osterborg A, Brandberg Y, Molostova V et al. Randomized, double-blind, placebo-controlled trial of recombinant human erythropoietin, epoetin Beta, in hematologic malignancies. *J Clin Oncol* 2002;20:2486-2494.

(8) Henke M, Laszig R, Ruebe C et al. Erythropoietin to treat head and neck cancer patients with anaemia undergoing radiotherapy: randomised, double-blind, placebo-controlled trial. *Lancet* 2003;362:1255-60.

(9) Aapro M, Leonard RC, Barnadas A et al. Effect of once-weekly epoetin beta on survival in patients with metastatic breast cancer receiving anthracycline- and/or taxane-based chemotherapy: results of the Breast Cancer-Anemia and the Value of Erythropoietin (BRAVE) study. *J Clin Oncol* 2008;26:592-598.

(10) Osterborg A, Boogaerts MA, Cimino R et al. Recombinant human erythropoietin in transfusion-dependent anemic patients with multiple myeloma and non-Hodgkin´s lymphoma - a randomized multicenter study. *Blood* 1996;87:2675-82.

(11) Ten Bokkel Huinink WW, De Swart CA, Van Toorn DW et al. Controlled multicentre study of the influence of subcutaneous recombinant human erythropoietin on anaemia and transfusion dependency in patients with ovarian carcinoma treated with platinum-based chemotherapy. *Medical Oncology* 1998;15:174-82.

(12) Cazzola M, Messinger D, Battistel V et al. Recombinant human erythropoietin in the anemia associated with multiple myeloma or non-Hodgkin´s lymphoma: dose finding and identification of predictors of response. *Blood* 1995;86:4446-53.

(13) Nitz U, Gluz O, Zuna I et al. Final results from the prospective phase III WSG-ARA trial: impact of adjuvant darbepoetin alfa on event-free survival in early breast cancer. *Ann Oncol* 2014;25:75-80.

(14) Strauss HG, Haensgen G, Dunst J et al. Effects of anemia correction with epoetin beta in patients receiving radiochemotherapy for advanced cervical cancer. *Int J Gynecol Cancer* 2008;18:515-24.

(15) Boogaerts M, Coiffier B, Kainz C, and the Epoetin beta QOL Working Group. Impact of epoetin beta on quality of life in patients with malignant disease. *Br J Cancer* 2003;88:988-995.

(16) Clinical Study Report MF4362 - Open-label, randomized phase III study to investigate the effect of epoetin beta on blood transfusion need in children with various malignant solid tumours treated with chemotherapy. Research Report - 1022737, August 16, 2006.

(17) Untch M, von MG, Konecny GE et al. PREPARE trial: a randomized phase III trial comparing preoperative, dose-dense, dose-intensified chemotherapy with epirubicin, paclitaxel, and CMF versus a standard-dosed epirubicin-cyclophosphamide followed by paclitaxel with or without darbepoetin alfa in primary breast cancer--outcome on prognosis. *Ann Oncol* 2011;22:1999-2006.
