# Supplementary material for: Does access to clinical study reports from the European Medicines Agency reduce reporting biases? A systematic review and meta-analysis of randomized controlled trials on the effect of erythropoiesis-stimulating agents in cancer patients
Source: PLoS One. 2017 Dec 11;12(12):e0189309. doi: 10.1371/journal.pone.0189309 (PMC5724886; doi:10.1371/journal.pone.0189309)
Supplement: S3 Table — CI, confidence interval; FACT-An, Functional Assessment of Cancer Therapy-Anemia; FACT-F, Functional Assessment of Cancer Therapy-Fatigue; MD, mean difference. (DOCX) [file pone.0189309.s012.docx]

**S3 Table: Meta-analyses for quality of life stratified by source of data**

|  | **Number of**  **comparisons** | **Number of**  **participants** | **Weight (%)** | **Effect estimate (95% CI)**  **random effects** | **P-value*** | **Effect estimate (95% CI)**  **fixed effects** | **P-value*** |
| --- | --- | --- | --- | --- | --- | --- | --- |
| **FACT-An 20** |  |  |  |  |  |  |  |
| Public domain | 8 | 1,561 | 59.1% | MD 5.51 (4.20, 6.82) |  | MD 5.51 (4.20, 6.82) |  |
| EMA documentation only | 6 | 978 | 40.9% | MD 0.20 (-1.93, 2.33) |  | MD 0.26 (-1.46, 1.98) |  |
| Total | 14 | 2,539 | 100% | MD 3.21 (1.38, 5.03) | **<0.001** | MD 3.59 (2.55, 4.63) | **<0.001** |
| **FACT-F 13** |  |  |  |  |  |  |  |
| Public domain | 18 | 4,965 | 82.0% | MD 2.37 (1.40, 3.35) |  | MD 2.08 (1.43, 2.72) |  |
| EMA documentation only | 5 | 806 | 18.0% | MD -0.12 (-1.63, 1.38) |  | MD -0.12 (-1.63, 1.38) |  |
| Total | 23 | 5,771 | 100% | MD 1.93 (1.04, 2.83) | **0.006** | MD 1.74 (1.15, 2.33) | **0.008** |
| **FACT-An Total** |  |  |  |  |  |  |  |
| Public domain | 11 | 2,284 | 66.9% | MD 5.97 (0.49, 11.44) |  | MD 5.62 (3.57, 7.68) |  |
| EMA documentation only | 6 | 1,270 | 33.1% | MD -0.17 (-4.82, 4.48) |  | MD -0.17 (-3.29, 2.96) |  |
| Total | 17 | 3,554 | 100% | MD 3.92 (-0.20, 8.04) | **0.09** | MD 3.88 (2.16, 5.60) | **0.002** |

* Test for subgroup differences between public domain versus EMA documentation only.

CI, confidence interval; FACT-An, Functional Assessment of Cancer Therapy-Anemia; FACT-F, Functional Assessment of Cancer Therapy-Fatigue; MD, mean difference.
