# Supplementary material for: Does access to clinical study reports from the European Medicines Agency reduce reporting biases? A systematic review and meta-analysis of randomized controlled trials on the effect of erythropoiesis-stimulating agents in cancer patients
Source: PLoS One. 2017 Dec 11;12(12):e0189309. doi: 10.1371/journal.pone.0189309 (PMC5724886; doi:10.1371/journal.pone.0189309)
Supplement: S4 Table — CI, confidence interval; FACT-An, Functional Assessment of Cancer Therapy-Anemia; FACT-F, Functional Assessment of Cancer Therapy-Fatigue; MD, mean difference. (DOCX) [file pone.0189309.s013.docx]

**S4 Table: Meta-analyses for quality of life stratified by source of data: sensitivity analyses with merged experimental arms for multi-arm studies**

|  | **Number of**  **comparisons*** | **Number of**  **participants** | **Weight (%)** | **Effect estimate (95% CI)**  **random effects** | **P-value**** |
| --- | --- | --- | --- | --- | --- |
| **FACT-An 20** |  |  |  |  |  |
| Public domain | 8 | 1,561 | 61.3% | MD 5.51 (4.20, 6.82) |  |
| EMA documentation only | 5 | 978 | 38.7% | MD 0.12 (-2.13, 2.36) |  |
| Total | 13 | 2,539 | 100% | MD 3.25 (1.38, 5.12) | **<0.001** |
| **FACT-F 13** |  |  |  |  |  |
| Public domain | 18 | 4,965 | 83.5% | MD 2.37 (1.40, 3.35) |  |
| EMA documentation only | 4 | 806 | 16.5% | MD -0.12 (-1.61, 1.41) |  |
| Total | 22 | 5,771 | 100% | MD 1.95 (1.04, 2.86) | **0.007** |
| **FACT-An Total** |  |  |  |  |  |
| Public domain | 10 | 2,284 | 68.0% | MD 6.48 (0.71, 12.25) |  |
| EMA documentation only | 5 | 1,270 | 32.0% | MD -0.66 (-5.58, 4.25) |  |
| Total | 15 | 3,554 | 100% | MD 4.03 (-0.34, 8.40) | **0.06** |

CI, confidence interval; FACT-An, Functional Assessment of Cancer Therapy-Anemia; FACT-F, Functional Assessment of Cancer Therapy-Fatigue; MD, mean difference.

* Experimental arms were merged for studies with ≥2 experimental arms.

** Test for subgroup differences between public domain versus EMA documentation only.
