# Supplementary material for: Does access to clinical study reports from the European Medicines Agency reduce reporting biases? A systematic review and meta-analysis of randomized controlled trials on the effect of erythropoiesis-stimulating agents in cancer patients
Source: PLoS One. 2017 Dec 11;12(12):e0189309. doi: 10.1371/journal.pone.0189309 (PMC5724886; doi:10.1371/journal.pone.0189309)
Supplement: S5 Table — CI, confidence interval; EMA, European Medicines Agency; FACT-An, Functional Assessment of Cancer Therapy-Anemia; FACT-F Functional Assessment of Cancer Therapy-Fatigue; Hb, hemoglobin; HR, hazard ratio; MD, mean difference; RBC, red blood cell; RR, risk ratio. (DOCX) [file pone.0189309.s014.docx]

**S5 Table: Direct comparison of pooled effect estimates from studies that reported discrepant data in the public domain and in EMA documentation**

|  |  | **Public domain** | | **EMA documentation** | |
| --- | --- | --- | --- | --- | --- |
|  | **Number of comparisons*** | **Number of participants** | **Random effect estimate (95% CI)** | **Number of participants** | **Random effects estimate**  **(95% CI)** |
| FACT-An | 2 | 476 | MD 4.25 (0.72, 7.78) | 504 | MD 4.28 (0.76, 7.81) |
| FACT-F | 2 | 476 | MD 3.56 (0.76, 6.35) | 505 | MD 3.49 (0.71, 6.27) |
| FACT-An Total 47 | 2 | 675 | MD 2.52 (-1.90, 6.94) | 981 | MD 2.52 (-1.50, 6.54) |
| Thrombovascular events | 9 | 3399 | RR 1.52 (1.09, 2.13) | 3403 | RR 1.22 (0.94, 1.58) |
| Hypertension | 3 | 487 | RR 1.88 (0.91, 3.88) | 487 | RR 2.00 (0.98, 4.09) |
| Overall survival | 12 | 5256 | HR 1.08 (0.98, 1.18) | 5253 | HR 1.06 (0.96, 1.17) |
| On-study mortality | 11 | 4819 | HR 1.21 (1.02, 1.43) | 4827 | HR 1.25 (1.04, 1.50) |
| Hematological response | 4 | 825 | RR 4.48 (3.39, 5.92) | 773 | RR 4.19 (2.97, 5.91) |
| Hb change | 20 | 4868 | MD 1.40 (1.11, 1.70) | 4898 | MD 1.32 (1.01, 1.62) |
| Participants receiving RBC transfusions | 5 | 982 | RR 0.78 (0.62, 0.99) | 946 | RR 0.64 (0.54, 0.75) |
| Number of RBC units transfused | 12 | 2083 | MD -0.70 (-1.11, -0.29) | 2146 | MD -1.11 (-1.75, -0.46) |

*Studies with multiple experimental arms were counted as separate comparisons

CI, confidence interval; EMA, European Medicines Agency; FACT-An, Functional Assessment of Cancer Therapy-Anemia; FACT-F Functional Assessment of Cancer Therapy-Fatigue; Hb, hemoglobin; HR, hazard ratio; MD, mean difference; RBC, red blood cell; RR, risk ratio.
