# Supplementary material for: Does access to clinical study reports from the European Medicines Agency reduce reporting biases? A systematic review and meta-analysis of randomized controlled trials on the effect of erythropoiesis-stimulating agents in cancer patients
Source: PLoS One. 2017 Dec 11;12(12):e0189309. doi: 10.1371/journal.pone.0189309 (PMC5724886; doi:10.1371/journal.pone.0189309)
Supplement: S6 Table — CI, confidence interval; EMA, European Medicines Agency; HR, hazard ratio. (DOCX) [file pone.0189309.s015.docx]

**S6 Table: Comparison of pooled effect estimates from studies where individual patient data were used for the analysis of on-study mortality and overall survival in previous analyses^1^ and estimates reported in EMA documentation**

|  |  | **Public domain** | | **EMA documentation** | |
| --- | --- | --- | --- | --- | --- |
|  | **Number of comparisons*** | **Number of participants** | **Random effect estimate (95% CI)** | **Number of participants** | **Random effects estimate**  **(95% CI)** |
| Overall survival | 11 | 3723 | HR 1.09 (0.99, 1.19) | 3701 | HR 1.07 (0.97, 1.18 |
| On-study mortality | 11 | 3723 | HR 1.20 (1.01, 1.42) | 3702 | HR 1.24 (1.03, 1.49) |

^1^ Bohlius J, Schmidlin K, Brillant C, Schwarzer G, Trelle S, Seidenfeld J, et al. Recombinant human erythropoiesis-stimulating agents and mortality in patients with cancer: a meta-analysis of randomised trials. Lancet, 2009;373: 1532–42.

*Studies with multiple experimental arms were counted as separate comparisons.

CI, confidence interval; EMA, European Medicines Agency; HR, hazard ratio.
